# Supplementary material for: Single-cell–level digital twins for preterm birth prevention strategies
Source: Sci Adv. 2026 Oct 21;12(43):eaed2741. doi: 10.1126/sciadv.aed2741 (PMC13693830; doi:10.1126/sciadv.aed2741)
Supplement: Supplementary file 1 [file sciadv.aed2741_sm.pdf]

Science Advances
AAAS

Supplementary Materials for

Single-cell–level digital twins for preterm birth prevention strategies

Jakob Einhaus *et al.*

Corresponding author: Brice Gaudilliere, gbrice@stanford.edu

Sci. Adv. **12**, eaed2741 (2026)
DOI: 10.1126/sciadv.aed2741

This PDF file includes:

Figs. S1 to S7
Tables S1 to S4

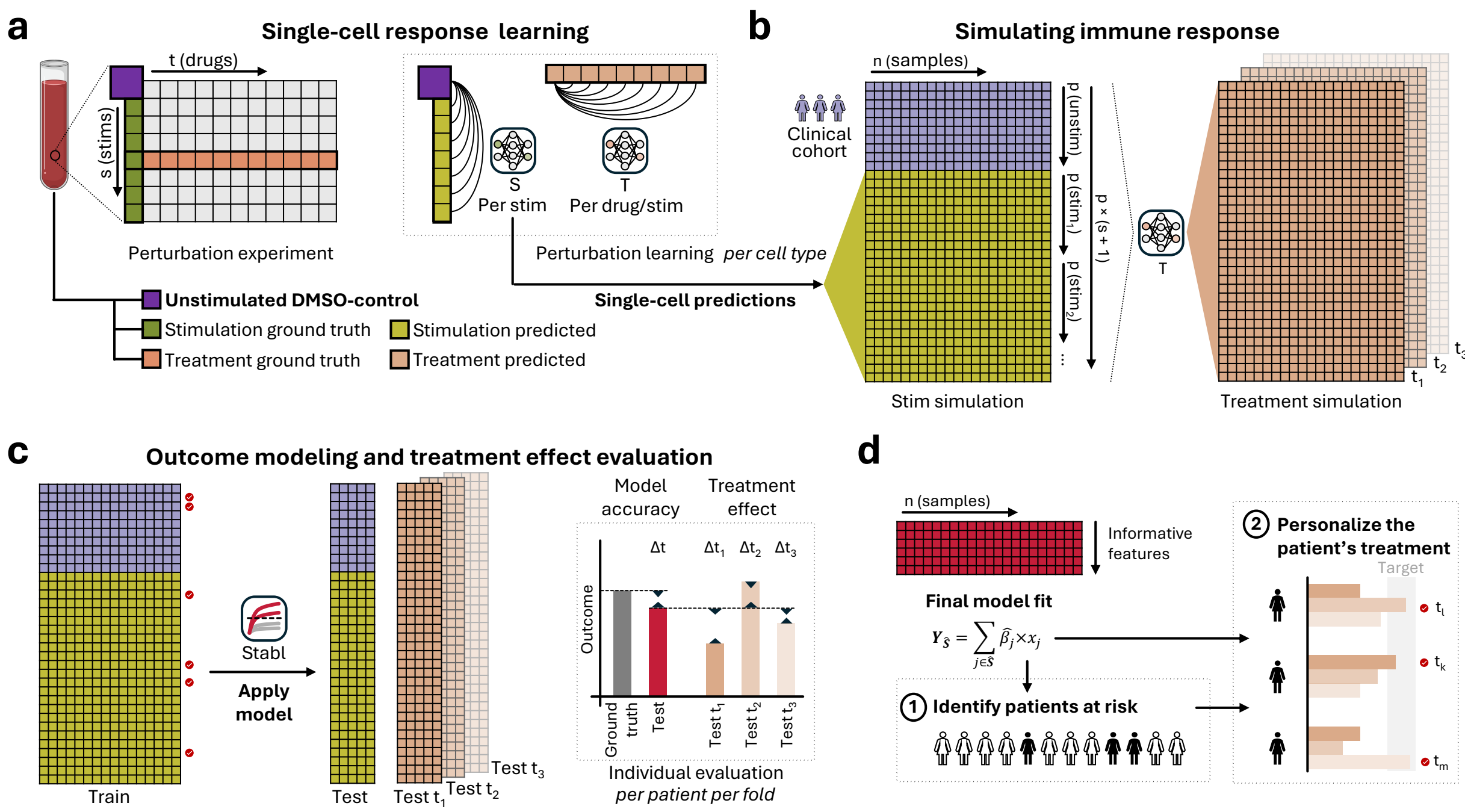

Figure S1 | Infographic depicting the SIMCO workflow in detail.

a. From a whole blood sample, perturbation experiments are performed to generate ground truth single-cell data (e.g., mass cytometry) over a matrix of s stimulations and t drug treatments. Per cell type, CellOT models S_v and T_k are trained to learn perturbation responses to each stimulation v and to each treatment k (within a specific stimulation condition).

b. From an independent clinical cohort, n baseline samples (i.e., unstimulated and unexposed to treatments) are analyzed with mass cytometry, providing p features per sample. The resulting baseline dataset is expanded to include v stimulation scenarios through CellOT single-cell predictions, resulting in a simulated dataset containing (v +1) x p features. Using the stimulation-specific T_k CellOT models, a final dataset is built simulating single-cell treatment responses for each stimulation condition. The simulated treatment-response dataset contains (v +1) x p x t features.

c. The Stabl algorithm is then used to build a multivariable model predictive of a given clinical outcome (e.g., the time to labor onset). The expanded dataset (before simulated treatment) is split into a training and testing cohorts for cross-validation. Stabl selects clinical outcome-informative features and builds a multivariable model on the training cohort. The resulting Stabl model is then applied to the testing cohort and its treatment-simulated datasets t_k. Model accuracy is evaluated as the difference between true and predicted outcome on the testing cohort. Treatment effects are evaluated per patient per cross-validation fold as the difference between the predicted outcome on the untreated data and the treatment-stimulated data.

d. The final Stabl model can be used to identify patients at risk for a given clinical outcome, and evaluation on the simulated-treatment responses can help personalize the patient's treatment.

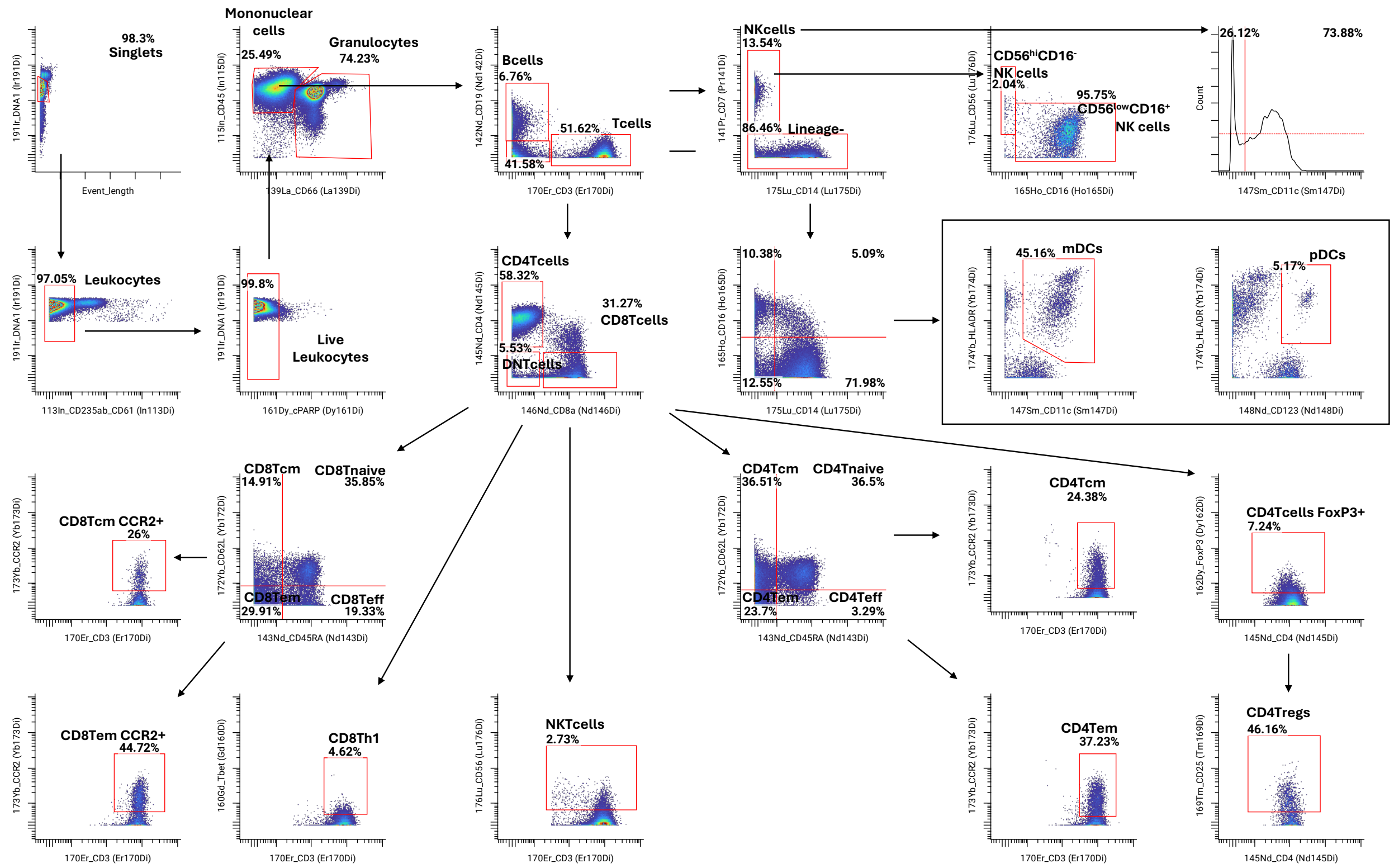

Figure S2 | Unified gating strategy for mass cytometry of the perturbation dataset and the clinical dataset by Stelzer et al. (33). A total of 28 cell populations was manually gated and used for the analysis.

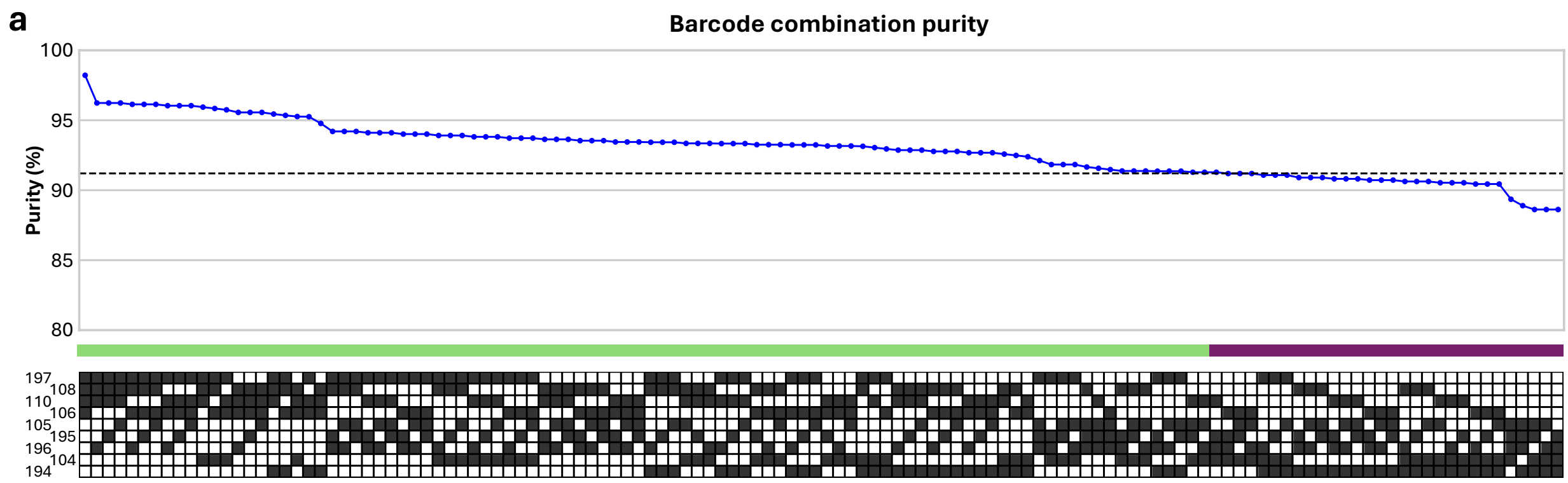

b

Isotope	Purity
Au197	99.9
Pd108	99.5
Pd110	99.4
Pd106	99.3
Pd105	98.8
Pt195	97.3
Pt196	97.3
Pd104	97.0
Pt194	96.5

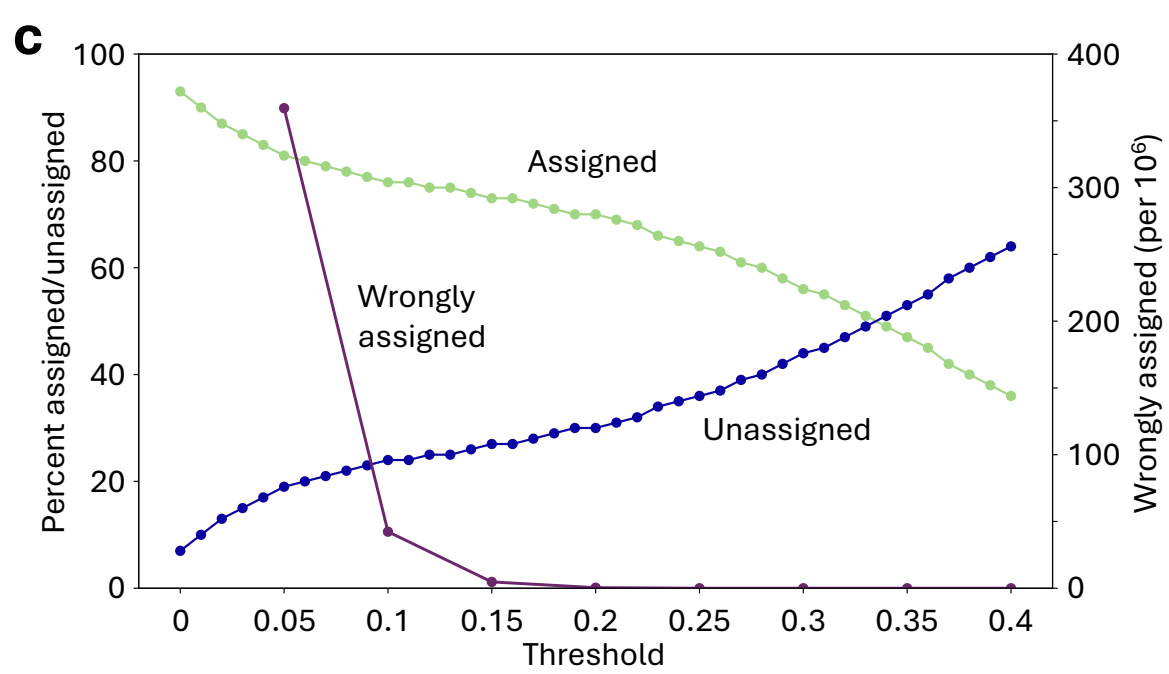

Figure S3 | Design of the 96-well barcoding scheme.
Palladium (Pd, 104, 105, 106, 108, 110), platinum (Pt, 194, 195, 196) and gold (Au, 197) isotopes were used to construct a nine-choose-four barcoding system.
a. The theoretical purity of each of 126 possible barcoding labels calculated as product of the individual isotopes' purities. The 96 purest barcodes (green) were used, while the 30 most impure barcodes (purple) served as control. The threshold between used and unused barcodes was a purity of 91.28 %.
b. Table of isotope purities based on the vendor's specifications.
c. Varying the debarcoding threshold demonstrated how lower thresholds achieve higher assignment rates, while assigning more cells to unused ("wrong") barcodes (purple).

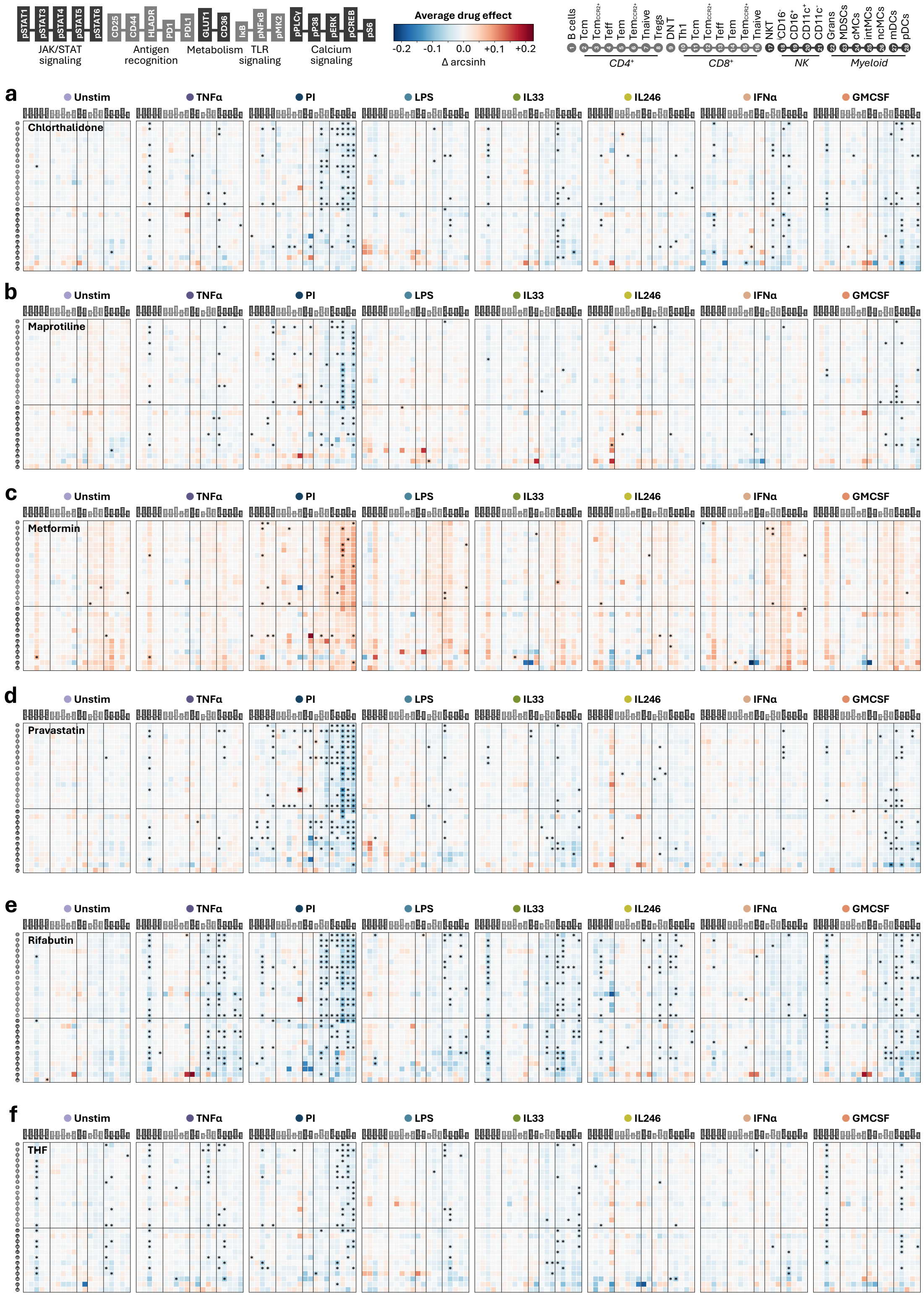

Figure S4 | Overview heatmaps of drug effects achieved by all drug candidates. Average drug effects across n = 11 pregnant individuals for chlorthalidone (**a**), maprotiline (**b**), metformin (**c**), pravastatin (**d**), rifabutin (**e**), and 5-methyl-tetrahydrofolate (THF, **f**). For SA, SALPZ, and lansoprazole see **Figure S5**.

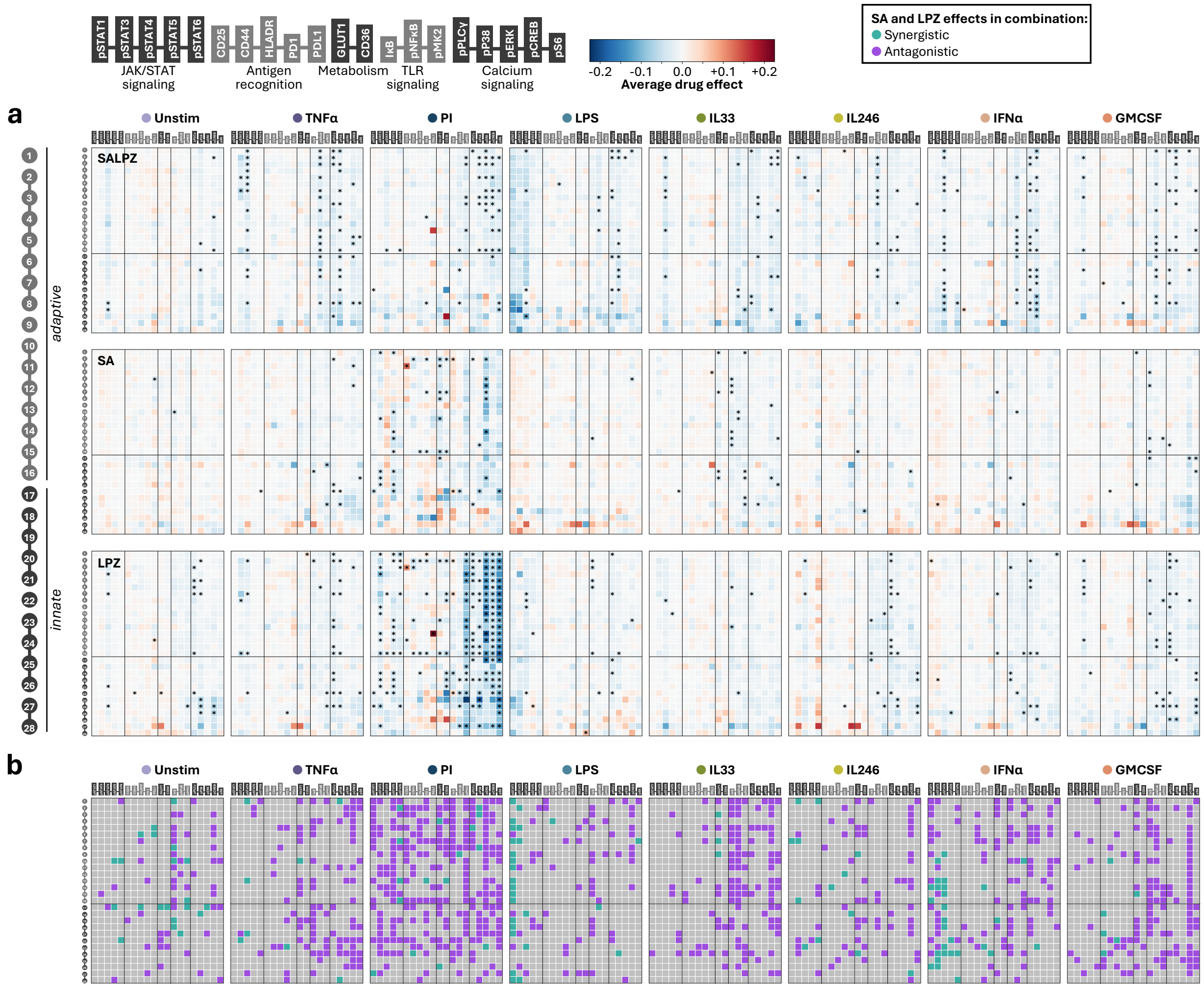

Figure S5 | Synergistic, additive, and antagonistic effects of SA, lansoprazole, and SALPZ.

a. Average drug effects mediated by SALPZ (top), SA (middle), and lansoprazole (LPZ, bottom) on n = 11 pregnant individuals.

b. Comparing the observed effects of SALPZ to the summed effects of SA and lansoprazole classified the effects as synergistic, additive, or antagonistic. Across all features, SALPZ effects were synergistic in 3%, additive in 79%, and antagonistic in 18%.

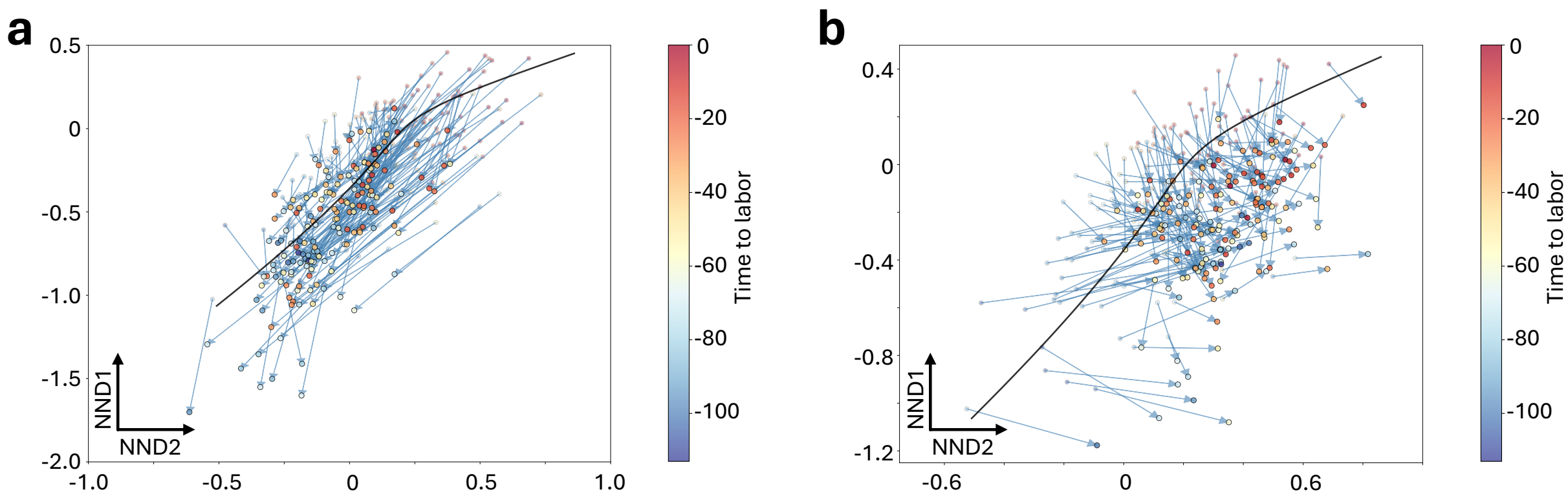

Figure S6 | Projection of drug effects in pseudotime. Multivariate, supervised pseudotime analysis using a neural network to encode time to labor organized samples coherently by gestational age in the latent space. Drug effects of the combination aspirin and lansoprazole (**a**) and chlorthalidone (**b**) are shown as arrows pointing from the pseudotime position of the untreated to the pseudotime position of the treated sample (n=177).

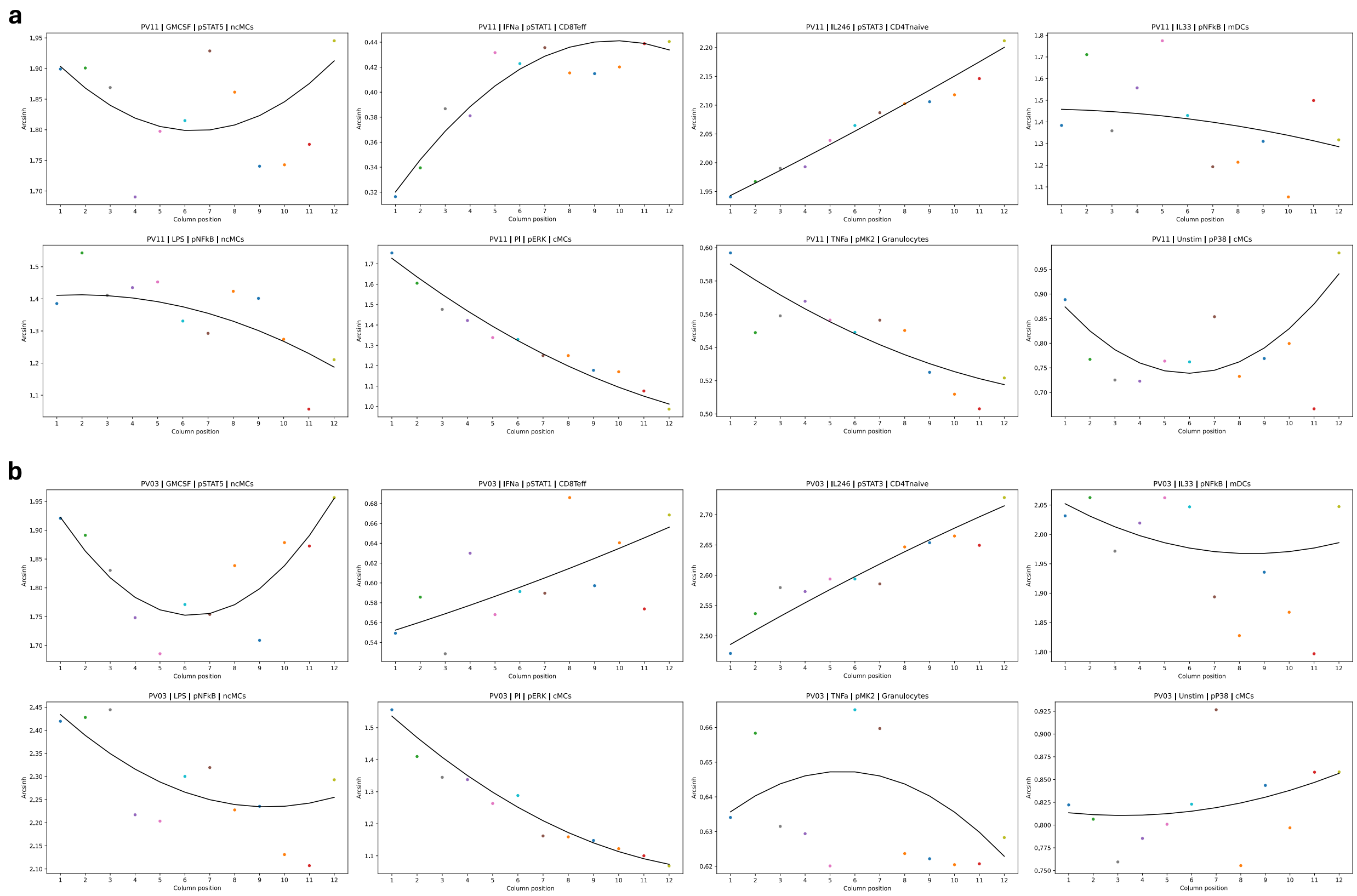

Figure S7 | Modeling of time-dependent effects on signaling response across columns. For individuals PV11 (**a**) and PV03 (**b**), the modeled time-dependent effects on signaling responses across columns is shown for eight example features. Per individual and per feature, a smooth spline was fitted across the feature values of all columns (k = 2, smoothing = 1.5, weight = 1 for drug-columns, weight = 2 for DMSO columns) to account for time-dependent effects in signaling response. Drug treatments were organized as depicted in Figure 2a. DMSO columns were placed in column 1, 6, and 12.

Target	Clone	Supplier	Catalog #
CD235ab	HIR2	Biolegend	306615
CD61	VI-PL2	BD	555752
CD45	HI30	Biolegend	304045
CD66	CD66a-B1.1	BD	551354
CD7	M-T701	BD	555359
CD19	HIB19	Biolegend	302247
CD45RA	HI100	Biolegend	304143
CD11b	ICRF44	Biolegend	301337
CD4	RPA-T4	Biolegend	300541
CD8a	RPA-T8	BD	557084
CD11c	Bu15	Biolegend	337221
CD123	6H6	Biolegend	306027
PD-1	D4W2J	CST	63815
CD44	IM-7	Biolegend	103065
CD36	D8L9T	CST	39914
PD-L1	E1L3N	CST	85164
GLUT1	EPR3915	Abcam	ab252403
CD16	3G8	Biolegend	302051
CD25	M-A251	Biolegend	356102
CD3	UCHT1	Biolegend	300443
CD62L	DREG.200	Thermo Fisher	BMS1015
CCR2	K036C2	Biolegend	357202
HLA-DR	L243	Biolegend	307651
CD14	M5E2	Biolegend	301843
CD56	NCAM16.2	BD	559043
pCREB	87G3	CST	9198
pSTAT5	C11C5	CST	51879SF
pp38	36/p38	BD	612281
pSTAT1	14/P-STAT1	BD	612132
pSTAT3	M9C6	CST	74309SF
pS6	D57.2.2E	CST	4858
pMAPKAPK2	27B7	CST	3007
Tbet	4B10	Thermo Fisher	14-5825-82
cPARP	F21-852	BD	552597
FoxP3	PCH101	Thermo Fisher	14-4776-82
IκB	L35A5	CST	4814
pNFκB	K10-895.12.50	BD	558393
pERK1/2	D13.14.4E	CST	45899SF
pSTAT6	A15137E	Biolegend	686002
pPLCγ2	A17025A	Biolegend	612402
pSTAT4	38/P-STAT4	BD	612739

Table S1 | Antibody panel of the perturbation atlas mass cytometry experiments.

Stimulation	Average MAE	Average MMD	Average Spearman R
GMCSF	0.07732349	0.01001739	0.81835463
IFNα	0.08200975	0.01234101	0.78866706
IL246	0.08949932	0.01130042	0.8079325
IL33	0.03352717	0.00247631	0.93522383
LPS	0.1246932	0.02581156	0.57759379
PI	0.14132662	0.02632193	0.72555276
TNFα	0.04773449	0.00462354	0.90259611

Table S2 | CellOT performance in cross-validation across stimulation conditions.

Model	AUROC	AUPRC	Pearson R	R2	Global RMSE	RMSE term	RMSE preterm
Stim (SIMCO) + Unstim (original)	0.750 [0.585, 0.879]	0.294 [0.117, 0.539]	0.645 [0.549, 0.728]	0.415 [0.283, 0.526]	24.120 [21.390, 26.705]	23.837	27.197
Stim (SIMCO) + Unstim (original) *GSS-LOPO*	0.735 [0.599, 0.863]	0.202 [0.097, 0.424]	0.595 [0.495, 0.686]	0.347 [0.203, 0.462]	25.486 [22.803, 28.170]	25.376	26.735
Stim (SIMCO) + Unstim (original) + EGA	0.894 [0.819, 0.960]	0.477 [0.239, 0.732]	0.947 [0.930, 0.962]	0.896 [0.862, 0.921]	10.158 [8.911, 11.371]	8.384	22.053
EGA	0.500 [0.500, 0.500]	0.079 [0.040, 0.119]	0.940 [0.915, 0.960]	0.834 [0.759, 0.894]	12.837 [10.499, 14.950]	7.369	38.092
Stim (original) + Unstim (original)	0.822 [0.728, 0.910]	0.262 [0.134, 0.521]	0.745 [0.668, 0.811]	0.551 [0.448, 0.645]	21.136 [18.788, 23.525]	21.437	17.249
Unstim (original)	0.709 [0.599, 0.807]	0.221 [0.082, 0.437]	0.399 [0.267, 0.517]	0.145 [0.024, 0.244]	29.161 [26.642, 31.806]	29.258	28.007
Stim (SIMCO)	0.745 [0.581, 0.884]	0.221 [0.104, 0.455]	0.635 [0.537, 0.722]	0.403 [0.273, 0.519]	24.376 [21.703, 26.942]	24.09	27.485
Stim (SIMCO) + Stim (original) + Unstim (original)	0.843 [0.748, 0.927]	0.322 [0.166, 0.598]	0.801 [0.735, 0.854]	0.621 [0.525, 0.693]	19.409 [17.270, 21.721]	19.576	17.334
Stim (SIMCO) + Stim (original) + Unstim (original) + EGA	0.927 [0.852, 0.980]	0.590 [0.340, 0.823]	0.952 [0.938, 0.964]	0.900 [0.872, 0.922]	9.957 [8.785, 11.058]	8.749	19.034

Table S3 | Predictive performances for simulated data by SIMCO, original data by Stelzer et al. (33), and estimated gestational age (EGA). Unstimulated features were always from the original dataset, while stim features were either simulated by SIMCO or extracted from the original data. A leave-on-preterm-out modified GroupShuffleSplit (GSS-LOPO) cross-validated model was included for comparison as well.

	Hyperparameters	Values
**Stabl grid search**	Base estimator	[Lasso, Alasso, EN]
	Final model fitted	[Linear regression, Random Forest, XGBoost]
	Artificial noise	[Knockoff, Random permutation]
	Noise proportion	[0.8, 1.0]
**XGBoost grid search (final layer)**	n_estimators	[300, 500]
	max_depth	[2, 4, 10]
	learning_rate	[0.01, 0.05]
	subsample	[0.5, 0.7]
	colsample_bytree	[0.5,0.8]
	gamma	[0, 1]
	reg_alpha	[0]
	reg_lambda	[1]

Table S4 | List of hyperparameter grid search for Stabl/XGBoost models.
